# Acute physical exercise improves shifting in adolescents at school: evidence for a dopaminergic contribution

**DOI:** 10.3389/fnbeh.2015.00196

**Published:** 2015-07-28

**Authors:** Timo Berse, Kathrin Rolfes, Jonathan Barenberg, Stephan Dutke, Gregor Kuhlenbäumer, Klaus Völker, Bernward Winter, Michael Wittig, Stefan Knecht

**Affiliations:** ^1^Psychology of Learning in Education and Instruction Group, Institute for Psychology in Education, Department of Psychology, University of MünsterMünster, Germany; ^2^Performance Physiology Group, Institute for Sports Medicine, Department of Medicine, University of MünsterMünster, Germany; ^3^Molecular Neurobiology Group, Department of Neurology, University of KielKiel, Germany; ^4^Department of Social Sciences, Catholic University of Applied Sciences North Rhine-WestphaliaMünster, Germany; ^5^Genetics and Bioinformatics, Institute of Clinical Molecular Biology, University of KielKiel, Germany; ^6^Preventive and Rehabilitative Neurology at Mauritius Hospital and Institute of Clinical Neuroscience and Medical Psychology, Heinrich Heine University DüsseldorfDüsseldorf, Germany

**Keywords:** physical exercise, acute intense exercise, executive functions, shifting, cognitive flexibility, adolescents, dopamine, gene

## Abstract

The executive function of shifting between mental sets demands cognitive flexibility. Based on evidence that physical exercise fostered cognition, we tested whether acute physical exercise can improve shifting in an unselected sample of adolescents. Genetic polymorphisms were analyzed to gain more insight into possibly contributing neurophysiological processes. We examined 297 students aged between 13 and 17 years in their schools. Physical exercise was manipulated by an intense incremental exercise condition using bicycle ergometers and a control condition which involved watching an infotainment cartoon while sitting calm. The order of conditions was counterbalanced between participants. Shifting was assessed by a switching task after both conditions. Acute intense physical exercise significantly improved shifting as indicated by reduced switch costs. Exercise-induced performance gains in switch costs were predicted by a single nucleotide polymorphism (SNP) targeting the Dopamine Transporter (DAT1/SLCA6A3) gene suggesting that the brain dopamine system contributed to the effect. The results demonstrate the potential of acute physical exercise to improve cognitive flexibility in adolescents. The field conditions of the present approach suggest applications in schools.

## Introduction

Physical exercise is not only capable of fostering physical health but also of improving brain functioning and cognitive processes (Hillman et al., 2008). Among cognitive processes, the executive functions of working memory (cf. Baddeley, 1996) were shown to especially benefit from physical exercise in an early meta-analysis (Colcombe and Kramer, 2003). Colcombe and Kramer reviewed studies with healthy older adults engaging in aerobic fitness training. In a recent review, Barenberg et al. (2011) also included studies on younger participants and distinguished chronic exercise and acute exercise on the intervention side. Chronic exercise studies implement activity programs involving repeated exercise sessions whereas acute exercise studies apply only a single exercise session. On the side of the executive functions, Barenberg et al. (2011) distinguished tasks demanding inhibition (of prepotent responses), shifting (between mental sets), and updating (of working memory content). The latter differentiation was adopted from the latent variable analysis by Miyake et al. (2000) who had shown that inhibition, shifting, and updating are moderately correlating but clearly separable executive functions. Barenberg et al. (2011) revealed that acute exercise can be beneficial for executive functioning measured after exercise. But consistent positive effects emerged only for inhibition performance. Updating was not studied so far, and shifting was shown to be unrelated to acute exercise in four studies. However, the generalizability of the latter studies may be questioned. Two of them investigated healthy adults in small samples with *N* < 19 (Tomporowski and Ganio, 2006; Coles and Tomporowski, 2008), one studied depressive adults (Kubesch et al., 2003) and one examined overweight children (Tomporowski et al., 2008). In contrast, two more recent studies demonstrated acute exercise effects on shifting in young adults (Berse et al., 2014; Barenberg et al., 2015). Thus, the evidence of acute exercise effects on shifting performance is still inconclusive. Moreover, no study examined this question in adolescents so far (Verburgh et al., 2014).

Behavioral neuroscience substantially adds to the field, because it provides methods to gain insight into possibly contributing processes on the neurophysiological level. So far, acute physical exercise was shown to stimulate a variety of physiological processes. It is supposed to elevate the monoamines norepinephrine, serotonine (Meeusen and Piacentini, 2001), epinephrine and dopamine (Winter et al., 2007), neurotrophic factors (Ferris et al., 2007; Rasmussen et al., 2009), and blood oxygenation (Ekkekakis, 2009; Rooks et al., 2010). Executive functions were shown to rely on a prefrontal cortex-basal ganglia network balancing cognitive flexibility and cognitive stability. The network is modulated by dopaminergic signaling. Baseline dopamine functioning seems to vary among individuals and differential responses to dopaminergic manipulations of the system were observed (Hazy et al., 2006; O'Reilly and Frank, 2006; Cools and D'Esposito, 2011). Combining the above mentioned neurophysiological findings reveals the fronto-striatal dopamine system underlying cognitive flexibility to be the most likely candidate to mediate between acute physical exercise and shifting. One study investigated the possible dopaminergic contribution to exercise effects on shifting in humans. Stroth et al. (2011) implemented a genetic marker of central dopamine availability in their study and found evidence for an involvement of dopamine in chronic exercise effects on shifting. Comparable approaches in acute exercise studies are missing.

Concerning further potential mediators, norepinephrine might also play a role. It seems to regulate arousal and cortical activity across different executive functions (Logue and Gould, 2014). This might argue for an unspecific involvement in acute exercise effects on shifting. Other unspecific effects could also be due to blood oxygenation. Epinephrine predicted inhibition performance during exercise in a study by McMorris et al. (2009). It is questionable, however, whether epinephrine also contributes to shifting after exercise. The nerve growth factor brain derived neurotrophic factor (BDNF) and the serotonine system did not account for shifting demands so far (Alfimova et al., 2012; Logue and Gould, 2014).

Against this background, the present study addressed the question whether acute physical exercise fosters shifting in adolescents. We hypothesized exercise to improve shifting performance in 13–17 year old adolescents, so that participants shift more efficiently between task sets after exercise compared to a control condition. It was expected that the dopamine system contributed to the effect. This was tested by the analysis of genetic polymorphisms. Genetic polymorphisms reflect individual differences in baseline neurophysiological functioning. If the fronto-striatal dopamine system contributed to acute physical exercise effects on shifting, the corresponding dopaminergic polymorphisms should predict differential effectiveness of the manipulation. Moreover, we explored the predictive value of further non-genetic individual difference variables.

## Materials and methods

### Participants

A total of 297 adolescents (158 male) took part in the study. Their mean age was 14.8 years (SD = 0.9; range = 13–17). According to the declaration of Helsinki, written informed consent was obtained from all students and their parents. Participation was voluntary and could be quit at any time of the experiment. The procedure had been approved by the ethical committee of the German Psychological Society. Twenty-nine participants failed to complete the study, mostly due to illness in at least one of the experimental sessions or due to concerns about sports capability.

### Materials and variables

#### Health screening

Sports physicians conducted a brief anamnesis and examination to ensure that participants were capable of attending intense physical exercise. They also assessed body mass index and waist-to-hip ratio. A self-developed health questionnaire was given to collect information about consumption of caffeine, cigarettes, alcohol, other drugs, and medication affecting the central nervous system. Furthermore, participants indicated their habitual physical activity on a self-developed five-point scale.

#### Fitness test

Participants underwent a field test running protocol on a marked track in the gym to assess individual fitness levels. They started running at a speed of 8 km/h. Every 3 min speed was enhanced by 2 km/h until exhaustion. Running speed was controlled by an acoustic signal indicating when to reach the next mark. Medically trained personal took capillary blood samples from the ear lobes at the beginning of the test, after each speed level and after 3 and 5 min recovery to diagnose blood lactate concentrations. Blood lactate analysis was done using a photometric method and a commercially available kit (EKF Diagnostic, Magdeburg, Germany). While capillary blood samples were collected, participants rated their perceived exertion on the Borg-Scale (Borg, 1970) ranging from 6 (no exhaustion at all) to 20 (complete exhaustion). Heart rate was continuously measured by a chest strap sensor (T31 coded system, Polar Electro, Germany). Individual speed at the anaerobic threshold represents a fitness indicator, which was employed as an individual difference variable and to check for the intensity of our exercise manipulation.

#### Physical exercise

Physical exercise was manipulated in two conditions, a control condition and an acute intense exercise condition. In the control condition, participants were instructed to watch an infotainment cartoon episode on human body functions on the computer screen while sitting in a relaxed position. The condition was designed to resemble an educational setting with low cognitive and physical load.

The intense exercise condition was chosen according to findings in recent reviews on the exercise-cognition relation. Chang et al. (2012) illustrated that the highest exercise intensities were most effective when cognitive performance was measured after a delay following exercise. Furthermore, Lambourne and Tomporowski (2010) showed that cycling interventions yielded larger effects on cognition than running protocols. So, participants performed two bouts of intense exercise on a bicycle ergometer (Ergo Bike Premium 8i, Daum Electronics, Germany). The intervention was based on an interval protocol described by Meyer et al. (1997). Participants aimed at cycling at a speed of 70 revolutions per minute (rpm) with a corridor of 60–80 rpm denoted acceptable. Pedaling resistance was raised by 25 Watt every 10 s. The procedure started with a 3 min warm up at 25 Watt. Then, the two bouts followed with a 3 min recovery at 25 Watt in between. Exercise bouts were terminated and recovery started when participants indicated exhaustion or cycling speed dropped below 60 rpm. The protocol ended with another 3 min recovery phase at 25 Watt.

The duration of treatments was comparable between the experimental conditions, with the exercise condition lasting approximately 10–14 min depending on individual performance. Physical activation was controlled by assessing blood lactate concentrations before and after both interventions. Furthermore, heart rate was continuously measured. Maximum heart rate at the end of the second exercise bout served as an indicator of willingness for exertion.

#### Shifting

We applied a switching task requiring predictable shifts between mental sets in alternating runs. Our task employing nonverbal stimuli (Baadte and Dutke, 2013) was a modification of the number-letter task (Rogers and Monsell, 1995), which proved to be a good indicator of shifting (Miyake et al., 2000). In the switching task, stimuli appear in a predictable clockwise sequence in the four corners of a computer screen. Presentation starts in the top left corner, continues in the top right corner and so on. Stimuli vary in two dimensions, color (blue or yellow) and shape (circle or triangle) in our modification of the Rogers and Monsell task. Participants use the same two buttons to decide on these stimuli. If a stimulus appears in the top half of the computer screen (top left corner or top right corner) participants indicate the shape with buttons representing circle and triangle. If a stimulus appears in the bottom half of the screen (bottom right corner or bottom left corner) they indicate the color with the same buttons now representing blue and yellow. The switching task encompasses a predictable switch between task sets in every second trial. All in all, in half of the trials a switch is demanded (top left and bottom right) and in the other half a given set is to be maintained (top right and bottom left). Switch trials differ from no-switch trials in that they require executive resources to a greater extent. Typically, this leads to higher latencies in switch trials compared to no-switch trials (Monsell, 2003). Switch costs are calculated as the speed and accuracy differences, respectively, between switch trials and no-switch trials. Participants completed 96 trials in six blocks (16 trials per block), with the first block serving as a practice block. Inter-stimulus interval was 1200 ms, maximum response time was 5000 ms.

#### Genetic polymorphisms

All genotypes were determined using genomic deoxyribonucleic acid obtained from white blood cells using standard procedures. Variable number tandem repeats (VNTRs) were genotyped using polymerase chain reaction amplification and manual scoring of the repeat numbers on high resolution agarose gel images. Single nucleotide polymorphisms (SNPs) were analyzed using a custom assay on a Sequenom mass array platform. Potentially relevant SNPs and VNTRs were analyzed. We included SNPs and VNTRs targeting the dopamine system (SNPs: rs6277, rs1800497, rs2283265, rs6280, rs936461, rs46000, rs37020, rs27072; VNTRs: 48bp-DRD4, 40bp-DAT1), the glutamate system (SNP: rs7301328), the serotonine system (SNPs: rs1352250, rs4570625), monoamines in general (SNP: rs6323), and BDNF (SNP: rs6265).

#### Mood valence and arousal

Participants rated their mood valence and arousal after the physical exercise conditions on the Self-Assessment Manikin (SAM; Lang, 1985), a nonverbal self-report measure of emotional state.

#### Intelligence

Two subtests (verbal retention and verbal processing capacity) of the German Berliner Intelligenzstruktur-Test [Berlin Intelligence Structure Test for gifted adolescents] (BIS-HB; Jäger et al., 2006) were given as measures of intelligence.

#### Attention deficit hyperactivity disorder (ADHD)

Occurrence of ADHD symptoms was self-assessed on the German Selbstbeurteilungsbogen für Aufmerksamkeitsdefizit-/Hyperaktivitätsstörungen [Self-Assessment of Attention Deficit Hyperactivity Disorder] (SBB-ADHS; Döpfner et al., 2008). The questionnaire consists of the subscales attention deficit, hyperactivity, and impulsivity.

### Design and procedure

We investigated the influence of the within-subjects factor physical exercise (control, exercise) on performance in the switching task. The order of conditions was counterbalanced between participants. Adolescents were randomly assigned to one of the orders (control-exercise or exercise-control). Except for the fitness test, which was conducted in the gym, the present study was run in a mobile laboratory, which was installed in a rebuilt motor coach. The mobile laboratory ensured a certain standard of experimental control in the field study. Participants were tested in groups of maximally 12 students. The procedure encompassed a pre-experimental session, two experimental sessions, and a post-experimental session. Sessions took place with a 1 week interval except for the post-experimental session, which was conducted 3 weeks after the second experimental session. The whole procedure took place during the regular school schedule and starting times were held constant within groups across sessions. Adolescents were instructed to abstain from caffeine and nicotine in preparation to the data acquisition.

In the pre-experimental session, participants did the health screening and filled in the health and ADHD questionnaires. They also underwent the fitness test and the switching task was instructed and practiced in a short version. The experimental sessions 1 and 2 were comparably structured. The treatment (control or exercise) was followed by the switching task (96 trials version). Blood lactate concentrations were measured before treatment (control or exercise), after treatment, and after the switching task. Valence and arousal were assessed after treatment. In the post-experimental session, participants accomplished the intelligence scales, and a venous blood sample was taken for gene analysis. Finally, participants got a personal fitness test evaluation for participation and they were debriefed.

## Results

### Preliminary analyses

#### Health screening

We used similar exclusion criteria as were used in the acute intense exercise study by Winter et al. (2007): daily consumption of more than five cups of coffee, more than nine cigarettes, or more than 50 g alcohol. Other predefined critical incidents were intake of other (illegal) drugs or medication affecting the central nervous system during the last month. Descriptive analysis revealed that 14 participants had to be excluded because of critical consumption of cigarettes (*n* = 2), cannabis (*n* = 5), methylphenidate (*n* = 4), and antihistamines (*n* = 3).

#### Shifting

Error rates were inspected to check for participants' understanding of the task and compliance to the experimental procedure. In accordance with our pilot studies, we observed high accuracy in both no-switch and switch trials (*M* > 95% correct responses). Extremely high frequencies of errors (higher than three times the interquartile range) were found in 27 participants. They were excluded from further analyses as it was reasonable to conclude that they didn't comply with the task. Finally, 227 participants formed the sample for hypothesis testing. Table 1 shows the descriptive statistics for accuracy and speed data of the switching task.

**Table 1:** **Speed and accuracy in the switching task by experimental conditions (***N*** = 227)**.

		**Condition**						
		**Control**		**Exercise**				
		**M**	**SD**	**M**	**SD**	***F***	***p***	**$\eta_{p}^{2}$**
Speed	No-switch	665.79	186.39	668.72	208.98	0.113	0.737	0.00
	Switch	859.72	275.75	847.66	287.14	1.284	0.258	0.01
	Costs	193.90	139.29	178.95	127.86	4.087	0.044	0.02
Accuracy	No-switch	98.18	1.96	98.20	1.75	0.022	0.883	0.00
	Switch	97.32	2.50	97.30	2.19	0.017	0.897	0.00
	Costs	0.86	2.77	0.90	2.32	0.042	0.837	0.00

*Speed is given in response latencies in milliseconds, accuracy in percentage of correct answers*.

#### Manipulation check

We inspected participants' blood lactate concentrations before and after treatment (control, exercise) and after the switching task to check whether our manipulation was effective. Table 2 shows that participants were physically active in the exercise condition and non-active in the control condition. Furthermore, lactate concentrations after treatment were contrasted to the lactate concentrations at the individual anaerobic threshold assessed in the fitness test. The pattern of differences revealed that in the control condition participants were below the lactate concentration measured at their anaerobic thresholds whereas in the exercise condition participants clearly outreached their thresholds. This pattern confirmed that exercise was done at a high intensity.

**Table 2:** **Blood lactate concentration (***N*** = 227)**.

	**Condition**			
	**Control**		**Exercise**	
	***M***	***SD***	**M**	***SD***
Before treatment	1.35	0.56	1.05	0.35
After treatment	1.07	0.40	10.19	2.76
After switching task	0.95	0.29	1.86	0.73
Contrast	−2.48	0.88	6.68	2.74

*Lactate concentration was measured in mmol/l. Contrast was calculated as the lactate concentration after treatment (control, exercise) subtracted by the lactate concentration at the individual anaerobic threshold in the fitness test*.

#### Genetic polymorphisms

Genetic polymorphisms [see Section Genetic polymorphisms (Materials and variables)] were successfully determined in a subgroup of the final sample (*N* = 131). Some school classes were unable to attend (parts of) the post-experimental session due to problems in the class schedule. All SNPs were tested for Hardy-Weinberg equilibrium. The SNP targeting monoamines in general (rs6323) was in disequilibrium and excluded from further analysis.

### Hypothesis testing

We conducted a 2 × 2 mixed factorial analysis of variance to test the effect of the within-subjects factor physical exercise (control, exercise) on performance in the switching task, controlling for the influence of the between-subjects factor order of conditions (control-exercise, exercise-control). The speed data revealed a significant effect of physical exercise on switch costs, *F*_(1, 225)_ = 4.09, *p* = 0.044, $\eta_{\text{p}}^{2}=0.02$, with lower costs after the exercise treatment compared to the control treatment (see Table 1). We did not observe significant differences with respect to the no-switch trials, *F*_(1, 225)_ = 0.11, *p* = 0.737, and switch trials, *F*_(1, 225)_ = 1.28, *p* = 0.258. With regard to the accuracy data, the main effect of physical exercise was non-significant for all three measures (all *F*_(1, 225)_ < 0.1).

**Figure 1:**
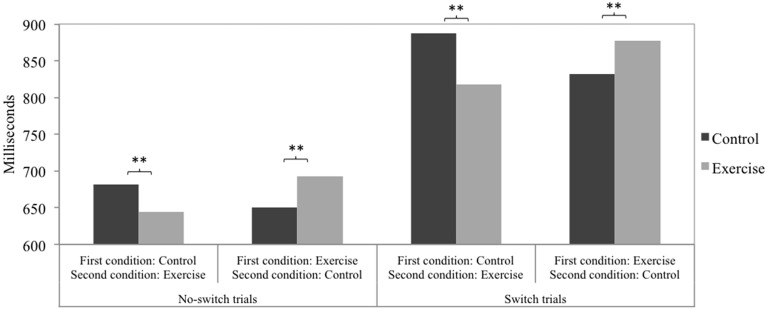
**The interaction of physical exercise (control, exercise) and order of conditions (control-exercise, exercise-control) on the speed measures of no-switch trials and switch trials**. *Post-hoc* tests were Bonferroni corrected. ^**^p < 0.01.

**Figure 2:**
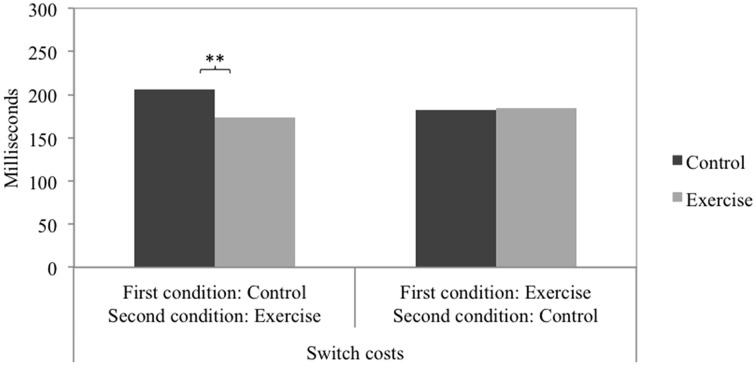
**The interaction of physical exercise (control, exercise) and order of conditions (control-exercise, exercise-control) on the speed measures of switch costs**. *Post-hoc* tests were Bonferroni corrected. ^**^p < 0.01.

Taking into account the between-subjects factor order of conditions yielded significant interactions of physical exercise (control, exercise) and order of conditions (control-exercise, exercise-control) on all three speed measures: switch costs [*F*_(1, 225)_ = 5.66, *p* = 0.018, $\eta_{\text{p}}^{2}=0.03$], no-switch trials [*F*_(1, 225)_ = 23.80, *p* < 0.001, $\eta_{\text{p}}^{2}=0.10$], and switch trials [*F*_(1, 225)_ = 28.09, *p* < 0.001, $\eta_{\text{p}}^{2}=0.11$]. Figures 1, 2 depict the pattern of the interaction effects. In the order control-exercise, reaction times were faster after the exercise treatment compared to the control treatment for both no-switch trials and switch trials (see Figure 1). In the order exercise-control, a reversed pattern was observed, with lower reaction times following the control treatment. Taken together, it becomes clear that no-switch and switch reaction times were lower in the second experimental session compared to the first experimental session. The numerically highest difference resulted for switch trials in the condition order control-exercise. Consequently, switch costs (see Figure 2) were lower after exercise when the exercise treatment was conducted in the second experimental session and the control treatment in the first. In the order exercise-control no significant differences emerged. The accuracy measures showed no significant interaction effects: switch costs [*F*_(1, 225)_ = 0.74, *p* = 0.391], no-switch trials [*F*_(1, 225)_ = 0.98, *p* = 0.324], and switch trials [*F*_(1, 225)_ = 3.30, *p* = 0.071]. No main effect of condition order was observed on any measure, neither speed nor accuracy.

### Analysis of genetic polymorphisms

Potential genetic predictors of the exercise effect were investigated by means of a multiple regression analysis. To obtain a measure for the performance gains after exercise, we subtracted performance after the exercise treatment from performance after the control treatment for the speed measures (no-switch trials, switch trials, and switch costs). The higher the value, the higher the exercise-induced gain. Switch trial gain was significantly higher than no-switch trial gain, *t*_(226)_ = 2.00, *p* = 0.047. Consequently, exercise-induced switch cost gain was positive. A stepwise regression procedure was run and normal distribution of the criterion variables was checked for as well as multicollinearity, homoscedasticity, and outliers of the predictors. We didn't find significant predictors of the no-switch trial gain. Table 3 shows the significant predictors for the switch cost gain and Table 4 displays the significant predictors for the switch trial gain.

**Table 3:** Regression of exercise-induced switch cost gain (***N*** = 131), ***b*** = unstandardized regression coefficient, ***SE*** = standard error, b^*^ = standardized regression coefficient.

	***b***	***SE***	b^*^	***t***	***p***
Intercept	4.04	12.49		0.32	0.747
DAT1/SLC6A3 rs46000	43.51	20.92	0.18	2.08	0.040

*Dopamine Transporter (DAT1)/SLC6A3 rs46000 is coded 0 = C/C, 1 = C/A or A/A. R^2^ for the model is 0.03, adjusted R^2^ is 0.03*.

**Table 4:** Regression of exercise induced switch trial gain (***N*** = 131), ***b*** = unstandardized regression coefficient, ***SE*** = standard error, b^*^ = standardized regression coefficient.

	***b***	***SE***	b^*^	***t***	***p***
Intercept	−38.36	22.56		−1.70	0.091
DAT1/SLC6A3 rs46000	81.01	32.09	0.21	2.52	0.013
DRD2/ANKK1 rs1800497	66.94	31.68	0.18	2.11	0.037

*rs46000 targeting the Dopamine transporter (DAT1) is coded 0 = C/C, 1 = C/A or A/A. rs1800497 targeting Dopamine D2 receptor density (DRD2)/ankyrin repeat and kinase domain containing 1 (ANKK1) is coded 0 = C/C, 1 = C/T or T/T. R^2^ for the whole model is 0.08, the corresponding adjusted R^2^ is 0.06*.

Switch cost gain was predicted by the DAT1/SLC6A3 (rs46000) polymorphism with carriers of the A allele benefitting more than homozygote C carriers. Switch trial gain was predicted by the DAT1/SLC6A3 (rs46000) polymorphism, with A carriers benefitting more than homozygote C carriers, and DRD2ANKK1 (rs1800497) polymorphism, with T carriers benefitting more than homozygote C carriers.

### Further analysis

Finally, further individual difference variables were analyzed in a multiple regression analysis predicting performance gains after exercise. Further predictors encompassed sex, age, body mass index, waist-to-hip ratio, self-reported habitual physical activity, valence and arousal after exercise, ADHD symptoms, intelligence, fitness, and willingness for exertion. Only one significant predictor resulted for no-switch benefits. No-switch benefits were predicted by intelligence [*b* = 2.71, *SE* = 0.78, b^*^ = 0.22, *t*_(1, 149)_ = 2.79, *p* = 0.006, $R_{adj}^{2}=0.04$].

## Discussion

The present study investigated the impact of acute physical exercise on shifting in 13–17 year old adolescents. Although there has been evidence that acute exercise has the potential to foster executive functioning across different age groups when tasks are conducted after exercise (e.g., Chang et al., 2012; Verburgh et al., 2014) we identified two open questions. Based on Miyake et al. (2000) and their finding that executive functioning is not a unitary concept but can be differentiated into separable executive functions, it has remained unclear whether exercise enhances specifically shifting performance. Additionally, studies focusing adolescents were, to our knowledge, lacking.

We expected adolescents to shift more efficiently between task sets after exercise compared to a control condition. Results were in accordance with our hypothesis. We observed significantly lower switch costs after exercise compared to the control condition (see Table 1). Although no significant differences were obtained in switch trials and no-switch trials, inspection of means in Table 1 and the significantly higher exercise-induced switch trial gain compared to the no-switch trial gain suggest that the reduction in switch costs could be mainly traced back to numerically lower reaction times in switch trials after exercise. Switch trials make higher demands on executive shifting performance than no-switch trials. Accordingly, our study demonstrated for the first time that acute exercise can foster shifting performance in adolescents and that this effect was specific for shifting demands rather than for non-shifting task demands, which were controlled for in no-switch trials.

Integrating the present experiment into former attempts to demonstrate acute exercise effects on shifting, it becomes clear that studies varied with regard to exercise mode and intensity. Both the present attempt and two recent studies in adults that yielded an effect so far (Berse et al., 2014; Barenberg et al., 2015) employed incremental and finally intense ergometer cycling (with aeorobic and anaerobic demands). In contrast, studies which failed to demonstrate this effect (Kubesch et al., 2003; Tomporowski and Ganio, 2006; Coles and Tomporowski, 2008) implemented moderate ergometer cycling. Tomporowski et al. (2008), who also did not find exercise effects on shifting, used a running protocol. In line with recent reviews (Lambourne and Tomporowski, 2010; Chang et al., 2012) the present work suggests that intense (compared to moderate) exercise as well as ergometer cycling (compared to running) is more likely to evoke executive benefits than other exercise interventions. Our findings are also in line with a recent meta-analysis which argued against age-specificity of acute exercise effects on executive functioning in general (Verburgh et al., 2014). Furthermore, the analysis of individual differences in the present study did not show any influence of age on the performance gains after exercise.

Another aim of the present study was to analyze genetic polymorphisms to gain more insight into possible contributing processes on the neurophysiological level. There is reason to assume that the dopamine system contributes to possible acute exercise effects on shifting though this has not been explicitly tested so far. In our study, switch cost gains were predicted by a polymorphism targeting the dopamine transporter DAT1/SLCA6A3 (rs46000) and switch trial gains were predicted by DAT1/SLCA6A3 (rs46000) and a polymorphism targeting dopamine D2 receptor DRD2/ANKK1 (rs1800497). We did not find genetic predictors for changes in no-switch performance. The results support the theory of a dopaminergic contribution to exercise-induced shifting gains. At least, this applies to the DAT1/SLCA6A3 polymorphism (rs46000) which predicted the switch cost gains, a measure that reflected improvements in executive rather than non-executive task demands.

The pattern of results matches findings from behavioral neuroscience. The dopamine transporter functions in the brain are region-specific with higher impact on subcortical rather than prefrontal areas. Concerning subcortical areas, the dopamine transporter releases and clears extracellular dopamine in the substantia nigra, in the striatum its main function is dopamine reuptake (Madras et al., 2005). The striatum is part of the fronto-striatal network described earlier which forms the basis of executive functioning. Two basic behavioral processes are balanced: Cognitive stability and cognitive flexibility (Cools and D'Esposito, 2011). Shifting, operationalized in the switching task, demands cognitive flexibility and the contribution of the striatum and the dopamine system to this process were recently outlined by Klanker et al. (2013). The dopamine transporter gene was associated with resting-state connectivity in the network which in turn predicted executive performance (Gordon et al., 2015). The fronto-striatal network underlying executive functioning was also shown to be sensitive to manipulations. This was demonstrated using dopaminergic agents (Cole et al., 2013; Costa et al., 2014). Even benefits in cognitive training of updating, which demands cognitive flexibility, were shown to be determined by striatal dopamine with training gains related to dopamine transporter polymorphisms (see the review by Bäckman and Nyberg, 2013). Exercise proved to elevate dopamine levels in humans (Winter et al., 2007) and in the striatum of rats (Hattori et al., 1994). As our exercise intervention resembled the one used by Winter et al. (2007) and switch cost gains in the present study were predicted by a dopamine transporter polymorphism, there is reason to conclude that acute, intense physical exercise fostered shifting performance by manipulating dopaminergic neurotransmission. More specifically, exercise seems to affect the fronto-striatal network by influencing striatal dopamine metabolism. We observed that carriers of the A allele of DAT1/SLCA6A3 polymorphism (rs46000) benefitted more from physical exercise than homozygote C carriers. Direct investigations are necessary at this point to reveal the exact underlying neurophysiological mechanism. We propose, however, tying in with Cools and D'Esposito (2011), that carriers of the A allele possess a suboptimal resting-state connectivity for cognitive flexibility antagonistically implying more suitable connectivity for cognitive stability. Recent results of Cummins et al. (2012) support this idea: The authors found reduced cognitive stability in an inhibition task in C carriers of the DAT1/SLCA6A3 polymorphism (rs46000). Summarized, resting-state connectivity of A carriers might be optimal for cognitive stability and suboptimal for cognitive flexibility (with C carriers behaving the other way around). Our results suggest that the possible suboptimal resting-state connectivity for cognitive flexibility in carriers of the A allele can be improved by physical exercise.

The DRD2/ANKK1 polymorphism (rs1800497) also exerts influence on dopaminergic signaling by affecting D2 receptor density in the striatum (Pohjalainen et al., 1998; Ritchie and Noble, 2003). It was an additional predictor of exercise-induced switch trial gains in the present study. As the switch trial performance is not adjusted for non-executive task demands, this indicator is a less specific measure for cognitive flexibility. Beyond the executive shifting demands, switch trials (in contrast to switch costs) comprise storage processes and the motor reaction amongst others. The motor reaction is also influenced by dopaminergic signaling and relies on striatal neurons (Kreitzer and Berke, 2011). This is why the DRD2/ANKK1 polymorphism (rs1800497) could not be compellingly related to executive shifting gains following exercise in the present study. However, the existing literature concerning this polymorphism suggests an involvement in cognitive flexibility (e.g., Stelzel et al., 2010; Markett et al., 2011; Wishart et al., 2011).

We did not find evidence for a contribution of the serotonin nor the glutamate system nor BDNF. Thus, our study demonstrated the involvement of the dopamine system in acute exercise effects on shifting rather than the involvement of other neurophysiological mechanisms.

The further analysis revealed only one significant non-genetic predictor. Performance gains in no-switch trials were predicted by verbal intelligence. Results indicated that more intelligent participants improved their no-switch performances after physical exercise to a greater extent than less intelligent participants. This finding was unexpected and replication and further analysis are desirable.

Regarding limitations of the present account, one might criticize the small effect size of the exercise effect limiting practical relevance. However, the size of the effect is not only influenced by the power of the intervention but also by the choice of the control condition, which is often a matter of debate in exercise experiments. Most often, unspecified resting conditions are implemented with the disadvantage that it is unclear what participants really do beyond being physically inactive. The control condition in our study, however, was specified in that participants were asked to attend to an infotainment cartoon episode resembling an everyday multimedia learning situation. However, this situation might have involved more activity than a resting and/or relaxation situation. Thus, our control condition implies a more conservative test for the exercise condition as it was shown that engaging in cognition activates behavioral and neural resources via dopaminergic networks (Boehler et al., 2011). So, the control condition might have had a beneficial effect on shifting as well and may have diminished the measured effect size. Another limitation might refer to the design. To control for the repeated measurements, the order of conditions was counterbalanced. Unexpectedly, the intervention interacted with the order of conditions. The exercise condition proved to be particularly effective when it was conducted in the second experimental session. This might be due to the lack of a baseline that could demonstrate the effectiveness of physical exercise in the first experimental session. Alternative explanations refer to a differential effectiveness of physical exercise in the time course of repeatedly conducting the switching task (which might reflect a learning process). This question should be examined in future studies as well (cf. Barenberg et al., 2015).

To sum up, the present study found a moderate positive effect of acute intense physical exercise on shifting performance and considerable evidence for a dopaminergic contribution to the effect in contrast to other neurophysiological explanations. From an application perspective, the findings encourage efforts to foster physical activity in adolescents. Even acute physical exercise lasting only 10–14 min proved to foster executive shifting performance, an important skill for academic achievement (Yeniad et al., 2013). The field conditions with the experimental procedure taking place in schools during the normal school schedule assure validity of the results for school-based interventions.

## Author contributions

SK, SD, KV, and BW designed and planned the study, KR and TB collected the data. Behavioral data was analyzed by TB and KR, gene data was analyzed by GK and MW. All authors took responsibility for interpreting the data. TB drafted the manuscript. All authors revised and approved the manuscript.

### Conflict of interest statement

The authors declare that the research was conducted in the absence of any commercial or financial relationships that could be construed as a potential conflict of interest.
