# Supplementary material for: Acute physical exercise improves shifting in adolescents at school: evidence for a dopaminergic contribution
Source: Front Behav Neurosci. 2015 Jul 28;9:196. doi: 10.3389/fnbeh.2015.00196 (PMC4517060; doi:10.3389/fnbeh.2015.00196)
Supplement: Supplementary file 1 [file Table1.DOCX]

Supplementary Material: Non-significant predictors in the analysis of genetic polymorphisms

Table 5

Regression of exercise-induced switch cost gain (*N* = 131).

|  | Beta ln | *t* | *p* |
| --- | --- | --- | --- |
| DRD2 rs6277 | .12 | 1.41 | .162 |
| DRD2/ANKK1 rs1800497 | .12 | 1.37 | .173 |
| DRD2 rs2283265 | .07 | .76 | .447 |
| DRD3 rs6280 | .13 | 1.50 | .137 |
| DRD4 rs936461 | .01 | .06 | .954 |
| DAT1 rs37020 | -.01 | -.15 | .878 |
| DAT1 SLC6A3 rs27072 | -.06 | -.63 | .530 |
| DRD4 48bp-VNTR | -.02 | -.17 | .864 |
| DAT1 40bp-VNTR | -.00 | -.05 | .960 |
| Glutamate NMDA rs7301328 | .16 | 1.81 | .072 |
| Serotonine TPH2 rs1352250 | .14 | 1.66 | .099 |
| Serotonine TPH2 rs4570625 | -.01 | -.09 | .926 |
| MAOA rs6323 | .04 | .48 | .630 |
| BDNF rs6265 | -.04 | -.45 | .656 |

Table 6

Regression of exercise-induced switch trial gain (*N* = 131).

|  | Beta ln | *t* | *p* |
| --- | --- | --- | --- |
| DRD2 rs6277 | .15 | 1.70 | .091 |
| DRD2 rs2283265 | .03 | .31 | .759 |
| DRD3 rs6280 | .11 | 1.34 | .183 |
| DRD4 rs936461 | .06 | .65 | .520 |
| DAT1 rs37020 | -.14 | -1.55 | .123 |
| DAT1 SLC6A3 rs27072 | -.07 | -.86 | .394 |
| DRD4 48bp-VNTR | .00 | .02 | .986 |
| DAT1 40bp-VNTR | -.01 | -.07 | .946 |
| Glutamate NMDA rs7301328 | .09 | 1.06 | .291 |
| Serotonine TPH2 rs1352250 | .04 | .52 | .606 |
| Serotonine TPH2 rs4570625 | -.03 | -.35 | .724 |
| MAOA rs6323 | .07 | .77 | .446 |
| BDNF rs6265 | -.05 | -.61 | .540 |
